# Double-trajectory lumbar screw placement guided by a set of 3D-printed surgical guide templates: a cadaver study

**DOI:** 10.1186/s12891-021-04149-0

**Published:** 2021-03-22

**Authors:** Yonghui Zhao, Jinlong Liang, Haotian Luo, Yongqing Xu, Sheng Lu

**Affiliations:** 1 Department of Orthopedics, The First People’s Hospital of Yunnan Province, The Affiliated Hospital of Kunming University of Science and Technology, The Key Laboratory of Digital Orthopedics of Yunnan Province, No. 157 Jinbi Road, Kunming, 650032 Yunnan China; 2 Department of Orthopedics, 920 Hospital of the Joint Logistic Support Force, 212 Daguan Road, Kunming, 650032 Yunnan China

**Keywords:** Cadaveric study, Operation guide template, Cortical bone trajectory screw, Pedicle screw

## Abstract

**Background:**

To improve the strength of posterior spine fixation in patients with osteoporosis, some scholars have proposed a method of simultaneously inserting traditional pedicle screws and cortical bone trajectory screws into the pedicle. However, due to the difficulty of the operation and few clinical applications, the safety and accuracy of this method are still unclear. The purpose of this study was to investigate the safety and accuracy of double-trajectory lumbar screw placement guided by surgical guide templates.

**Methods:**

Six wet lumbar specimens were selected for computed tomography (CT) scanning, a three-dimensional (3D) model of the lumbar spine was established using computer software, and surgical guide templates for double-trajectory [traditional pedicle trajectory (TPT) and cortical bone trajectory (CBT)] lumbar screw placement at various segments of the lumbar spine were designed and printed using a 3D printer. Screw placement was guided only by the surgical guide template, with no fluoroscopy. Postoperative CT examination was performed to determine whether the screw penetrated the screw path and the location and depth of penetration of the cortex. The preoperative and postoperative sagittal and axial angles of CBT screws or TPT screws were also measured and compared.

**Results:**

Four screws were placed in each vertebral body of six lumbar specimens for a total of 120 screws. Screw grades: 99 screws as grade 0, 15 as grade 1, six as grade 2, and zero as grade 3. Thus, grade 0 accounted for 82.5% of the screws. No significant differences in the preoperative and postoperative angles of the screws were found (*P* > 0.05).

**Conclusions:**

3D-printed surgical guide templates for double-trajectory screw placement can reduce the difficulty of surgery and the use of intraoperative fluoroscopy. Using such templates is a safe, feasible, and accurate screw placement method.

## Background

The pedicle screw technique is currently the main method of posterior lumbar fixation. However, for patients with osteoporosis, the fixation strength is significantly reduced, and loosening, pulling out, and breakage of screws are likely to occur after surgery, possibly leading to surgical failure [1–4]. To improve screws’ holding strength, Santoni et al. [5] increased the interface between the screw and the cortical bone by changing the trajectory of the screw and thus proposed cortical bone trajectory (CBT) screw technology. Different studies have confirmed that the fixation strength of CBT screws is superior to that of pedicle screws [5–7], but some scholars have suggested that CBT screws may not be able to provide strong fixation in orthopedic spine surgery, and traditional pedicle trajectory (TPT) and CBT screws began to be simultaneously placed in the pedicle during orthopedic spine surgery [8]. However, double screw fixation is difficult, and few related studies are available, which presents a considerable challenge for most surgeons. Therefore, in this study, individualized surgical guide templates for double-trajectory screw placement were designed with computer software and applied to cadaveric specimens to investigate their safety and accuracy.

## Methods

The study conformed to the tenets of the Declaration of Helsinki and was approved by the Ethics Committee of our hospital. Six formalin-fixed adult lumbar spine specimens were provided by the Department of Anatomy of Kunming Medical University, four of which were collected from males, while two were collected from females; their ages ranged from 42 to 67 years, with an average of 53.7 ± 9.0 years. Preoperative computed tomography (CT) was performed to exclude lumbar deformity, fractures, etc. The CT scan parameters were as follows: slice thickness 0.625 mm, voltage 120 kV, current 150 MA, and matrix 512 × 512.

### Preparation of the surgical guide template

**Fig. 1:**
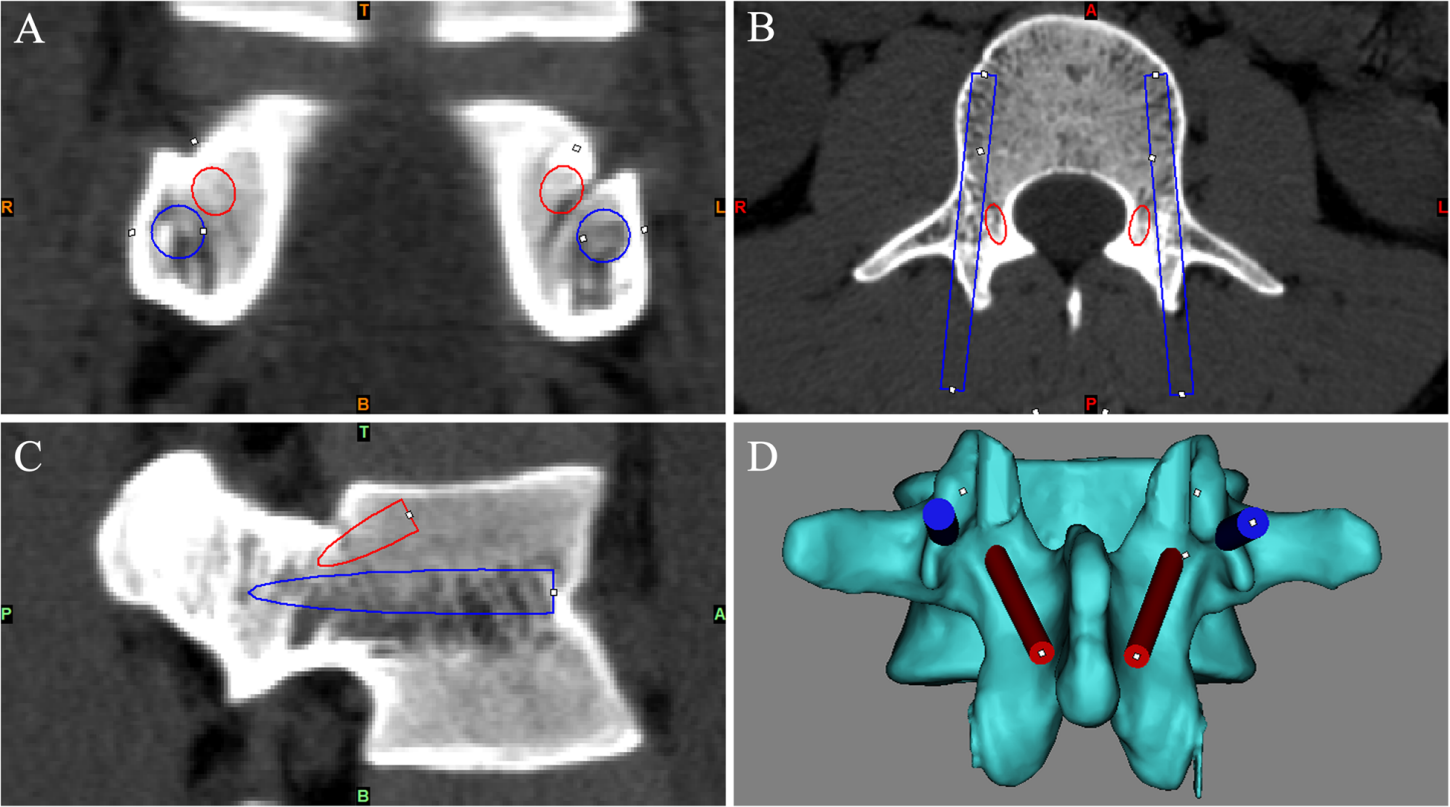
Design of the two screw paths in the same pedicle with Mimics19.0 software. The positions of the two screw paths were adjusted on the coronal **a**, axial **b**, and sagittal **c** planes. The diameter of the screw path was adjusted according to the size of pedicle such that the two screw paths were completely enclosed in the pedicle and did not intersect while the safety of the entry point for screw placement was ensured **d**

Before surgery, the lumbar spine model was established by Mimics 19.0 software based on CT data. Double-trajectory screw were designed in the same pedicle. Adjust the position of the two screw paths on the three-dimensional view, and the diameter of the screw path was adjusted according to the size of pedicle such that the two screw paths were completely enclosed in the pedicle with no overlap while ensuring the safety of the screw entry point (Fig. 1).

**Fig. 2:**
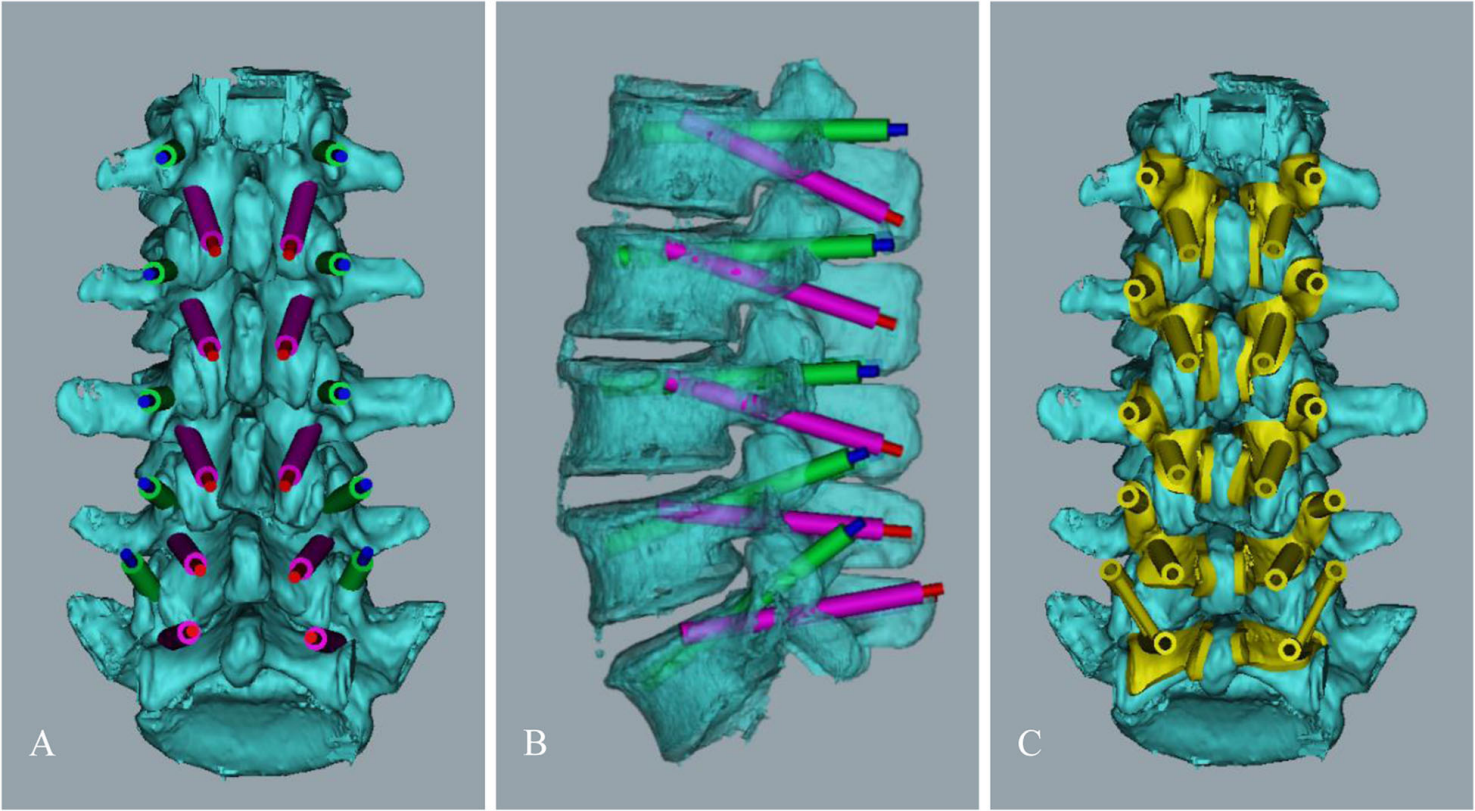
Anteroposterior view **a** and lateral view **b** of the lumbar spine after the design of the two screw paths with Mimics19.0 software. Use Geomagic studio12.0 software to extract the surface anatomical data of the region encompassing the spinous process and vertebral plate, and construct the attachment surface of the guide plate. Accurately register screw path with attachment surface to generate a virtual surgical guide **c**

The designed screw path is saved in SLE film, and use Geomagic studio12.0 software to extract the surface anatomical data of the region encompassing the spinous process and vertebral plate, and construct the attachment surface of the guide plate. Accurately register screw path with attachment surface to generate a virtual surgical guide (Fig. 2). Finally, select photosensitive resin materials to print out surgical guides through a 3D printer.

### Surgical technique

**Fig. 3:**
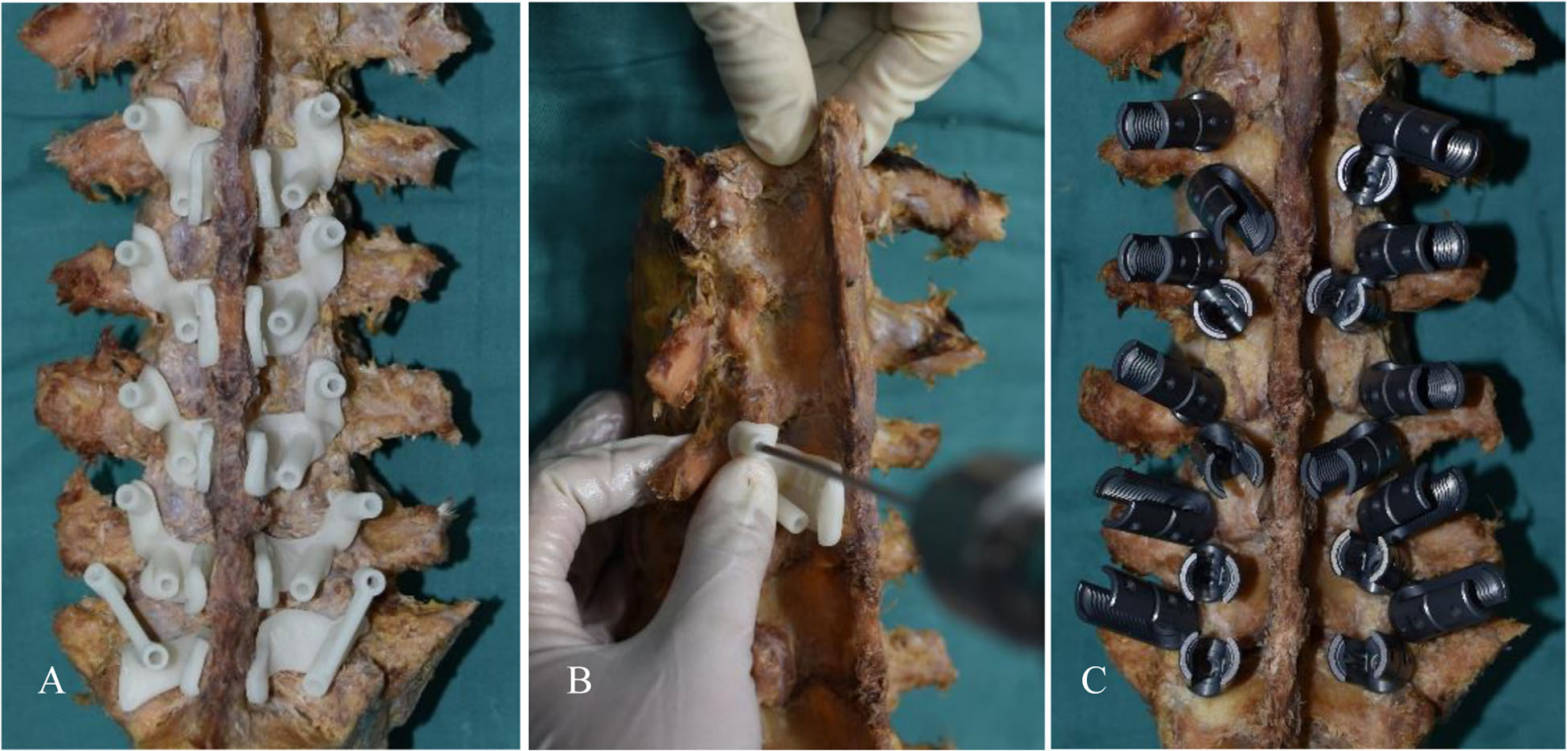
Each surgical guide template was tightly attached to the corresponding bone surface **a**; while an assistant helped to stabilize the specimen and the guide template, the surgeon used a drill bit to slowly drill along the axis of the screw path. After drilling for 20 mm–30 mm, a spherical probe was used to detect whether the screw path was complete. A tap drill was used for tapping, and the screws were placed **b**; the same method was used to place each screw **c**

A specimen was placed on the operating table, and the soft tissues, such as dorsal muscles and ligaments of the lumbar specimen, were dissected to fully expose the bony surface structures of the spinous processes, vertebral plates, and facet joints. Each surgical guide template was tightly attached to the corresponding bone surface. While an assistant stabilized the guide template, the surgeon used a drill bit (3.0-mm diameter) to slowly drill along the axis of the screw paths (inner diameter 3.2 mm). After drilling for 20–30 mm, a spherical probe was used to detect whether the screw path was complete. Two spherical probes were then used simultaneously to determine whether the two screw paths intersected. Then, a tap drill was used for tapping, and appropriately sized screws were placed. The same method was used to place each screw (Fig. 3). No fluoroscopy was performed during the screw placement process.

### Postoperative evaluation

**Fig. 4:**
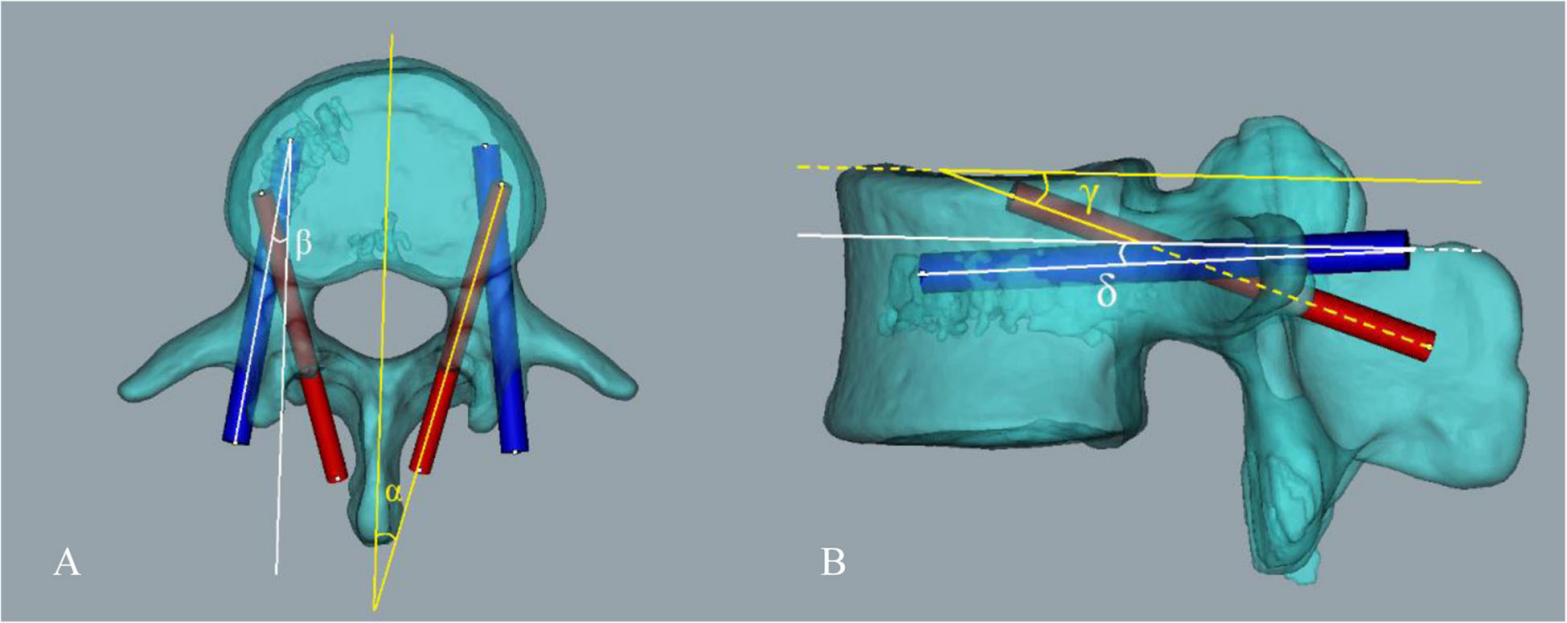
**a** Schematic diagram for measurement of axial angle α of the CBT screw and axial angle β of the TPT screw: α is the angle between the axis of the CBT screw and the midline of the vertebral body in the axial view; β is the angle between the axis of the TPT screw and the midline of the vertebral body in the axial view. **b** Schematic diagram for measurement of sagittal angle γ of the CBT screw and sagittal angle δ of the TPT screw: γ is the angle between the axis of the CBT screw and the upper endplate of the vertebral body on the sagittal plane; δ is the angle between the axis of the TPT screw and the upper endplate of the vertebral body on the sagittal plane

Postoperative screw placement was observed in axial and sagittal CT scans. According to Learch and Wiesner’s classification method, screw placement results were divided into four grades [9, 10]. The screw is completely in the cortical bone as grade 0. When the shortest distance from the most distal end of the screw to the adjacent cortical bone is less than 3 mm as grade 1, 3-6 mm as grade 2, and greater than 6 mm as grade 3. In addition, double-trajectory screw invasion of cortical bone wall at the same time may lead to pedicle fracture or loss of fixation force, so this condition is considered as grade 3. Additionally, the preoperative and postoperative sagittal and axial angles of the TPT and CBT screws in the transverse and sagittal planes were measured and compared (Fig. 4). The safety and accuracy of double-trajectory screw placement under the guidance of surgical guide templates were comprehensively evaluated.

### Statistical analysis

SPSS21.0 statistical software was used for analysis. The Kolmogorov-Smirnov (K-S) test judged the normal distribution of data. The data that normal distribution are expressed as $\overline{x}\pm s$. Independent sample t-test was used to compare the corresponding parameters before and after surgery, α < 0.05 was considered to be statistically significant.

## Results

**Fig. 5:**
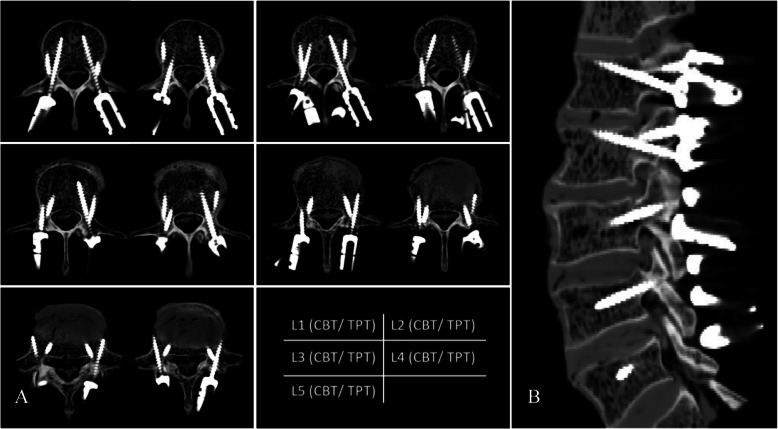
Lumbar CT scan showing that the positions of the TPT and CBT screws were satisfactory on the transverse plane **a** and the sagittal plane **b** of the L1-L5 vertebral body segments

A total of 30 guide templates were designed for the six specimens, and 20 screws were placed in each vertebral body specimen for a total of 120 screws. The TPT screws had a diameter of 5.0–6.0 mm and a length of 50–55 mm, and the CBT screws had a diameter of 4.0–5.0 mm and a length of 35–40 mm. Postoperative CT scans of the transverse and sagittal planes were performed to determine screw placement (Fig. 5). The results showed that 26 screws penetrated the screw path, with no simultaneous penetration of the screw paths by the TPT and CBT screws. Screw grades: 99 screws as grade 0, 15 as grade 1, six as grade 2, and zero as grade 3. Thus, grade 0 accounted for 82.5% of the screws (Table 1). The parameters measured before and after surgery are listed in Table 2. No significant differences in the sagittal and axial angles of the CBT screws or TPT screws at each lumbar segment were found (*P* > 0.05).

**Table 1:** Results of screw placement in various lumbar spine segments

Lumbar vertebral body	Screw placement results				Grade 0 (%)
	Grade 0	Grade 1	Grade 2	Grade 3	
L1	17	4	3	0	70.8%
L2	19	4	1	0	79.2%
L3	20	4	0	0	83.3%
L4	22	1	1	0	91.7%
L5	21	2	1	0	87.5%
Total	99	15	6	0	82.5%

**Table 2:** Comparison of the various parameters measured before and after surgery

Lumbar vertebral body	Sagittal angle of the CBT screws (°)		T-value	*P*-value	Axial angle of the CBT screws (°)		T-value	*P*-value	Sagittal angle of the TPT screws (°)		T-value	*P*-value	Axial angle of the TPT screws (°)		T-value	*P*-value
	Preoperative	Postoperative			Preoperative	Postoperative			Preoperative	Postoperative			Preoperative	Postoperative		
L1	20.6 ± 1.1	19.9 ± 1.0	1.646	0.114	25.7 ± 0.9	24.9 ± 1.4	1.484	0.152	3.4 ± 0.8	3.2 ± 0.9	0.497	0.624	18.4 ± 2.7	19.3 ± 2.7	− 0.830	0.415
L2	17.9 ± 1.0	18.2 ± 1.4	− 0.534	0.599	21.7 ± 1.7	21.4 ± 1.6	0.465	0.646	3.3 ± 0.9	3.6 ± 0.9	−0.914	0.371	15.3 ± 3.2	14.6 ± 2.1	0.658	0.519
L3	19.2 ± 0.9	18.9 ± 0.9	1.004	0.326	20.9 ± 2.7	21.2 ± 2.1	−0.242	0.811	5.1 ± 0.6	4.9 ± 0.7	0.555	0.585	15.4 ± 1.0	15.0 ± 1.0	0.817	0.423
L4	17.3 ± 1.5	16.9 ± 1.4	0.827	0.417	25.6 ± 1.2	23.6 ± 3.5	1.935	0.066	11.2 ± 1.4	10.9 ± 1.5	0.366	0.718	18.6 ± 2.6	19.9 ± 2.2	−1.246	0.226
L5	19.5 ± 0.6	20.1 ± 1.0	− 1.710	0.101	27.9 ± 1.5	27.4 ± 1.2	0.926	0.365	11.0 ± 1.3	11.5 ± 1.5	− 0.985	0.335	19.9 ± 2.7	21.9 ± 2.6	−1.867	0.075

## Discussion

With the aging of the population, osteoporosis has become a recognized health problem worldwide. At present, more than 200 million patients have osteoporosis in China, representing the largest osteoporosis patient population in the world. Osteoporosis patients are prone to screw loosening after surgery, which may lead to fixation failure. Osteoporosis has always been a troubling issue for spine surgeons. Different scholars have attempted to redesign screws to improve the screw holding force. Increasing the diameter and length of the screw can potentially produce larger pullout forces but may also increase the risk of fracturing the surrounding fragile bone [11, 12]. Compared with that of standard cylindrical screws, the effectiveness of the design of tapered pedicle screws in osteoporotic specimens has been controversial [11, 13, 14]. Mummaneni et al. [15] proposed a double-threaded screw, and the pullout test showed no significant difference compared with standard pedicle screws. In osteoporotic specimens, expansion screws have better pullout strength than standard screws [16–18]. However, these screws have not been promoted and widely applied, which may be related to the greater requirements for the screw material and the difficulty of removal after loosening. Bone cement screws can significantly increase the holding power of the screws, but a risk of leakage exists, and the high heat generated by polymerization can easily cause nerve tissue damage [19, 20]. CBT screws increase the contact area of cortical bone by changing the screw trajectory to improve the holding force, which has achieved satisfactory results in clinical applications [21–24]. However, some scholars believe that CBT screws may not be sufficient to provide strong fixation in spinal orthopedic surgery. To maximize the stability of correction, TPT screws and CBT screws were placed in the pedicle at the same time, and satisfactory results were obtained [8]. Despite adequate preoperative planning, pedicle splitting still occurred during the procedure, resulting in failed screw placement; however, such splitting did not have a substantial impact on the postoperative orthopedic outcome. Jeffrey et al. [25] used computer simulation to simultaneously place screws in two trajectories. The results showed that the success rate of screw placement was 34.1–79.6%, with significant differences among segments. Double-trajectory screw placement is technically difficult and high risk, and few relevant studies are currently available.

The purpose of this study was to design surgical guide templates for double-trajectory screw placement using computer software and to use them to guide the placement of screws in cadaveric specimens to further investigate the safety and accuracy of individualized surgical guide templates for double-trajectory screw placement. Before surgery, CT scanning of the specimens was performed to reconstruct 3D models of the lumbar spine and simulate double-trajectory screw placement. When simulating screw placement, screws with a diameter of 4.0–5.0 mm were first inserted according to the TPT and CBT. Upon confirmation that the screw entry point was safe and feasible, the positions of the TPT and CBT screws were adjusted on the 3D tomography image. According to the size of the pedicle, the diameter of the screws was gradually adjusted such that the screws were completely enclosed in cortical bone and the TPT and CBT screws did not intersect. The design of surgical guide templates for double-trajectory screw placement was successfully completed for all six specimens. The diameters of the screws selected for different segments differed, and differences existed within the same segment among The results show that grade 0 accounted for 82.5% of the screws (70.8% ~ 91.7%). In addition, the sagittal and axial angles of the CBT screws and TPT screws were measudifferent specimens. The selected pedicle screws were 5.0–6.0 mm in diameter and 50–55 mm in length; the CBT screws were 4.0–5.0 mm in diameter and 35–40 mm in length. The placement of all screws was guided by the surgical guide templates, and 120 screws were placed. The accuracy of screw placement was evaluated postoperatively based on 3D CT scans. Red preoperatively. No significant differences were found in the corresponding parameters of each lumbar spine segment before and after surgery (*P* > 0.05). Thus, 3D-printed surgical guide templates are safe and feasible for assisting double-trajectory lumbar screw placement and have high accuracy.

Notably, the following convenience will affect screw placement accuracy. On the one hand, for the design and manufacture of surgical guides, including the error caused by the surface contour of the reconstructed vertebral body may affect the accuracy of the attachment surface of the template; the size of the attachment surface is critical for the stability of the guide template. Increasing the attachment surface area of the guide template can improve stability but requires dissection of more soft tissue, which may increase intraoperative bleeding and the operative time [26]. The inner diameter and length of the navigation tube of the guide template may also affect the stability and accuracy of screw placement. We usually set the inner diameter to 3.2 mm, select a 3.0-mm drill bit, and select a navigation tube with a length of 25–35 mm; errors can occur during 3D printing, including those produced when printing the guide template after the STL data are imported to the 3D printer and those caused by shrinkage of the printed material during curing. On the other hand, in the screw placement process, the stability of the guide template can be a factor. Sufficient dissection of the soft tissues in the region where the guide template attaches is necessary such that the guide template can tightly and stably attach to the bone surface; the micromotion of the drill bit during drilling can result in errors to some extent compared with the preoperatively designed screw trajectory.

In terms of limitations of this study, the attachment surfaces of guide templates for double-trajectory screw placement cover the spinous processes and vertebral plates, thus encompassing an area larger than that of surgical guide templates for single-trajectory screw placement, which may require more intraoperative exposure, increase intraoperative bleeding, and prolong operation times. Because current evaluation methods for screw placement are not completely suitable for double-trajectory screw placement, this may lead to some bias in the results. We did not conduct further controlled studies of freehand screw placement. In addition, the sample size of this study was small, which may have some impact on the study results, and more studies with larger sample sizes are needed.

## Conclusion

3D-printed surgical guide templates for double-trajectory screw placement can reduce the difficulty of surgery and the use of intraoperative fluoroscopy. Using such templates is a safe, feasible, and accurate screw placement method.

## Data Availability

The datasets used and/or analyzed in the current study are available from the corresponding author upon reasonable request.

## Abbreviations

CT: Computed tomography
3D: Three-dimensional
CBT: Cortical bone trajectory
TPT: Traditional pedicle trajectory
STL: Stereolithograph
